# Gut Microbiome-Modified Polyphenolic Compounds Inhibit α-Synuclein Seeding and Spreading in α-Synucleinopathies

**DOI:** 10.3389/fnins.2020.00398

**Published:** 2020-05-04

**Authors:** Tritia R. Yamasaki, Kenjiro Ono, Lap Ho, Giulio M. Pasinetti

**Affiliations:** ^1^Department of Neurology, University of Kentucky, Lexington, KY, United States; ^2^Division of Neurology, Department of Internal Medicine, School of Medicine, Showa University, Tokyo, Japan; ^3^Department of Neurology, Icahn School of Medicine at Mount Sinai, New York, NY, United States

**Keywords:** α-synuclein, aggregation, microbiome, polyphenol, Parkinson’s disease, multiple system atrophy

## Abstract

Misfolding, aggregation and deposition of α-synuclein (α-syn) are major pathologic characteristics of Parkinson’s disease (PD) and the related synucleinopathy, multiple system atrophy (MSA). The spread of α-syn pathology across brain regions is thought to play a key role in the onset and progression of clinical phenotypes. Thus, there is increasing interest in developing strategies that target and attenuate α-syn aggregation and spread. Recent studies of brain-penetrating polyphenolic acids, namely, 3-hydroxybenzoic acid (3-HBA), 3,4-dihydroxybenzoic acid (3,4-diHBA), and 3-(3-hydroxyphenyl)propionic acid (3-HPPA) that are derived from gut microbiota metabolism of dietary polyphenols, show *in vitro* ability to effectively modulate α-syn misfolding, oligomerization, and mediate aggregated α-syn neurotoxicity. Here we investigate whether 3-HBA, 4-hydroxybenzoic acid (4-HBA), 3,4-diHBA, or 3-HPPA interfere with α-syn spreading in a cell-based system. Using HEK293 cells overexpressing α-syn-A53T-CFP/YFP, we assessed α-syn seeding activity using Fluorescence Resonance Energy Transfer (FRET) to detect and quantify α-syn aggregation. We demonstrated that 3-HPPA, 3,4-diHBA, 3-HBA, and 4-HBA significantly attenuated intracellular α-syn seeding aggregation. To determine whether our compounds could inhibit brain-derived seeding activity, we utilized insoluble α-syn extracted from post-mortem MSA or PD brain specimens. We found that 3-HPPA effectively attenuated MSA-induced aggregation of monomer into high molecular weight aggregates capable of inducing intracellular aggregation. Outcomes from our studies suggest interactions between gut microbiome and certain dietary factors may form the basis for effective therapies that modulate pathologic α-syn propagation. Collectively, our findings provide the basis for future developments of probiotic, prebiotic, or synbiotic approaches for modulating the onset and/or progression of α-synucleinopathies.

## Introduction

Parkinson’s disease (PD) and multiple system atrophy (MSA) involve abnormal aggregation of the protein α-synuclein (α-syn) (Papp et al., 1989; Spillantini et al., 1998a, b). The progression of α-syn pathology in neuronal cell bodies Lewy bodies (LBs) or Lewy neurites (LNs) in these neurodegenerative conditions is thought to involve transcellular spread of aggregation-prone forms of α-syn, throughout the neuraxis (Frost and Diamond, 2010; Guo and Lee, 2014). Some studies hypothesize initiation of α-syn misfolding in peripheral sites such as the olfactory bulb or enteric plexus of the gastrointestinal tract that subsequently propagates to the brain (Hawkes et al., 2007; Shannon et al., 2012). Animal models of gut-injected α-syn recombinant fibrils result in spread of α-syn pathology to the brain over time and vagotomy potentially modulates future development of PD-related symptoms and pathology (Svensson et al., 2015; Uemura et al., 2018; Kim et al., 2019). α-syn pathology has been found in sites as remote as the submandibular gland and peripheral nerves of the skin and colonic submucosa (Beach et al., 2010; Shannon et al., 2012; Adler et al., 2014, 2016; Donadio et al., 2014), implying that accumulation of pathologic forms of α-syn is not solely a central nervous system based process. Given potential cell-to-cell propagation of aggregation-prone forms, there is increasing interest in developing interventional strategies that will attenuate α-syn spread through interference with abnormal aggregation.

There is also evidence that α-syn may assemble into different fibrillar forms and that distinct conformations inherent to misfolded forms of α-syn may underlie different clinical presentations seen, especially in regard to the synucleinopathies, PD and MSA (Bousset et al., 2013; Peelaerts et al., 2015; Prusiner et al., 2015; Woerman et al., 2015; Peng et al., 2018; Yamasaki et al., 2019). The existence of different conformations of the α-syn aggregated state becomes important especially in light of therapies targeting α-syn aggregation.

Emerging evidence suggests that gut microbiota dysbiosis is a risk factor for developing α-syn pathology. A number of studies have documented the presence of dysbiosis in patients with PD suggesting that this is a risk factor for development of PD (Keshavarzian et al., 2015; Scheperjans et al., 2015; Heintz-Buschart et al., 2018). Moreover, recent preclinical observations support a cause-and-effect relationship between gut microbiota dysbiosis and PD pathophysiology (Erny et al., 2015; Klingelhoefer and Reichmann, 2015; Sampson et al., 2016). In particular in mice with neuronal overexpression of a wild-type human α-syn protein, colonization with fecal microbiota from PD patients significantly promoted PD-type pathophysiology while colonization via healthy donor fecal microbiota did not develop a similar phenotype (Sampson et al., 2016). Moreover, there is evidence suggesting that gut microbiota contribute to PD pathology by disrupting interactions between the enteric nervous system and the brain (Klingelhoefer and Reichmann, 2015). Recently, it has been reported that functional bacterial amyloid proteins may promote cross-seeded aggregation of α-syn, amyloid β-protein, tau, and others to initiate prion-like propagation (Friedland et al., 2020). On the other hand, gut microbiota may contribute to PD pathophysiology by metabolizing dietary compounds such as dietary fibers into short-chain fatty acids that are essential in promoting brain microglia maturation, leading to a heightened neuroinflammatory response which may contribute to PD pathophysiology (Sampson et al., 2016). Phenolic compounds modulate the gut-brain axis, which transform these phenolic compounds into physiologically active and neuroprotective compounds through the gut microbiome**-**metabolism (Reddy et al., 2020). These metabolites may exert their neuroprotective effects in neurodegenerative diseases such as PD and Alzheimer’s disease (AD) (Reddy et al., 2020). We recently observed that interpersonal heterogeneity in gut microbiota may lead to interpersonal variabilities the efficacy to metabolize dietary flavanols into select biologically available phenolic acid metabolities (Ho et al., 2019). Preclinical investigation by our group has demonstrated that dietary supplementation with select bioactive polyphenol-rich dietary preparations, such as a select grape seed polyphenol extract (GSPE) and a standardized Bioactive Dietary Polyphenol Preparation (BDPP, comprised of a select Concord grape juice, GSPE and resveratrol) are mechanistically effective in modulating diverse neuropathologic phenotypes (Frolinger et al., 2019). Following GSPE supplementation, we observed brain accumulations of select polyphenol metabolites in the form of monophenolic acids, such as 3-hydroxybenzoic acid (3-HBA) and 3-(3-hydroxyphenyl)propionic acid (3-HPPA), that are generated by gut microbiota fermentation of GSPE. Furthermore, previous work by our group has demonstrated treatment with a flavanol rich preparation (FRP) in gnotobiotic mice yields brain bioavailable polyphenol metabolites including the monophenolic compounds, 4-HBA, 3,4-dihydroxybenzoic acid (3,4-diHBA), and 3-HPPA (Pasinetti et al., 2018). We demonstrated that three of the 15 biologically available FRP-derived metabolites identified in cecum specimens of gnotobiotic mice, namely 3,4-diHBA, 3-HBA, and 3-HPPA, accumulate in the brain although 12 phenolic acid metabolites were detectable in plasma specimens from the mice (Ho et al., 2019). We found that these three brain-available phenolic acids are bioactive in modulating α-syn misfolding *in vitro*. Moreover, we also demonstrated that bioactive phenolic acids effectively modulate the development to PD-type neuropathy and behavioral phenotypes in a drosophila model of α-synucleinopathy (Ho et al., 2019).

## Methods

### Reagents

3-HBA, 4-HBA, 3,4-diHBA, and 3-HPPA (Ho et al., 2019; Supplementary Figure S1) were obtained commercially from Sigma-Aldrich (United States). Monomeric synthetic α-syn was purchased from rPeptide (Watkinsville, GA, United States).

### Human Brain Specimens

The Movement Disorders Center Neuropathology Core, Washington University, St. Louis, provided clinically and neuro-pathologically well-characterized postmortem frozen brain tissue (Table 1; Cairns et al., 2015). Post mortem brains were collected from 50% male/female subjects at ages ranging from 59 to 86 years of age. Routinely, microscopy was performed on the left hemibrain and biochemistry was performed using the right hemibrain. α-syn-immunoreactive inclusion bodies were observed only in PD and MSA utilizing phosphor-dependent anti-α-syn immunohistochemistry (Cell Applications, San Diego, CA, United States) and #64 (Wako, Osaka, Japan). Frozen tissue was prepared as previously described (Bagchi et al., 2013). Tissue was first dissected into small pieces (~1 mm square) using a scalpel and a cutting board cooled with solid CO_2_. Tissue was then weighed and serially extracted with a series of buffers, using a dounce homogenizer (Kontes) for homogenization. A ratio of 3 mL buffer to 1 g of tissue was maintained throughout the extraction process. Initial homogenization was performed in high salt buffer (50 mM Tris-HCl, pH 7.4, 750 mM NaCl, 5 mM EDTA) with protease inhibitors (Sigma), followed by centrifugation at 100,000 × g for 20 min at 4°C. Supernatant was removed and used as the buffer “soluble” fraction. The pellet was homogenized in 1% Triton X-100 in high salt buffer with protease inhibitors, followed by centrifugation at 100,000 × g for 20 min at 4°C. The pellet was extracted with 1M sucrose and 1% Triton X-100 in high salt buffer. The myelin component and supernatant were removed, and the pellet was then washed twice with Tris-buffered saline (TBS), resuspended in TBS, with protease inhibitor (Sigma), aliquoted, and frozen at –80°C and used as the detergent “insoluble” fraction.

**TABLE 1:** Clinical and neuropathological characteristics of brain samples for α-synucleinopathy.

**Sample diagnosis**	**Seeding activity**	**Sample area**	**Disease duration (years)**	**Post mortem interval (h)**	**Primary diagnosis**	**Additional diagnoses**	**BraaK LB stage (0–6)**	**Braak NFT stage (I-VI)**	**Amyloid stage (A–C)**
PD 1	Low	AC	17	5	DLBD	ADNC, TDP-MTL	6	III	C
PD 2	Low	AC	22	4	DLBD	ADNC	6	I	C
PD 5	High	AC	12	13	DLBD	0	5	I	0
MSA 1	Low	BG CB	13	16	MSA*	ADNC	0	II	B
MSA 4	High	BG	8	5	MSA*	ADNC	0	I	A

PD, Parkinson’s disease; MSA, mutiple system atrophy; AC, anterior cingulate; BG, basal ganglia; CB, cerebellum; DLBD, diffuse lewy body disease; *, MSA-nigrostriatal type; ADNC, Alzheimer disease neuropathologic change; TDP-MTL, TDP-43 proteinopathy in medial temporal lobe.

### α-Syn Aggregation

Recombinant α-syn was resusupended in aggregation buffer (100 mM NaCl, 20 mM TrisHCl, pH 7.4) to generate a 446 μM stock. This was further diluted in aggregation buffer to final concentration of 10 μM in final volume of 200 μl. Aggregation in the presence or absence of phenolic acid compounds at a molar ratio of 10:1 phenolic acid compound: α-syn monomer concentration was used. For experiments utilizing human brain, 5 μL of insoluble fraction, sonicated for 3 min at 65 A on a Qsonica 700 (Newtown, CT, United States) at 4°C, was added to each reaction mixture, for a final volume of 200 μl per reaction mixture. Aggregation was initiated by shaking at 1,500 rpm at 37°C for 24 h in an Eppendorf Thermomixer C (Hauppauge, NY, United States).

### Fluorescence Resonance Energy Transfer (FRET Assay)

Monoclonal biosensor cells (stable cell line 1H α-syn A53T-CFP/YFP) made as previously described (Yamasaki et al., 2019) were plated in 96-well plates at 20,000 cells per well and grown overnight. Aggregated α-syn formed in the presence or absence of phenolic compounds was introduced into the cells at various concentrations (i.e., 2, 10, 350 nM, as specified in experiments below) as calculated from the initial monomeric concentration. This was done in the presence of Lipofectamine reagent (Invitrogen, Waltham, MA, United States) in reaction volumes of 20 μl/reaction. Samples were then added dropwise to wells (total volume per well 150 μl). Technical quadruplicates were performed for each sample. Unless otherwise specified, incubation with cells was for 24–72 h, depending on the experiment. All experiments involving brain lysate were incubated for 24 h. Cells were then trypsinized and transferred to 96-well round-bottom plates, fixed in 4% paraformaldehyde (Electron Microscopy Services, Hatfield, PA) in PBS for 10 min, then centrifuged and resuspended in flow buffer (1 mM EDTA and 1% FBS in HBSS). Cell aggregate load was quantified using a MACSQuant VYB (Miltenyi, Auburn, CA, United States) as previously described (Holmes et al., 2014). Analysis was performed using FlowJo v10 software (TreeStar, Ashland, OR, United States).

### Statistics

All analyses, unless otherwise noted, were performed using Prism software (Graphpad). Student *t*-tests were performed with *post hoc* Dunnett corrections for the number of comparisons per set. *p*-values of < 0.05 were considered to be statistically significant. Graphs are displayed as mean ± SEM.

## Results

### Gut Microbiome-Modified Phenolic Acid Compounds Interfere With Formation of Aggregation-Prone Forms of Recombinant α-Syn

Based on the efficacy of 3-HBA, 3,4-diHBA and 3-HPPA to interfere with aggregation in prior studies, we extended our current investigation into a cell-based model of α-syn aggregation and tested the ability of gut microbiota derived phenolic compounds to interfere with α-syn aggregation derived from α-syn derived from PD and MSA brain fractions.

To determine whether the compounds 3-HBA, 4-HBA, 3,4-diHBA, and 3-HPPA interfere with aggregation of α-syn monomer, we incubated 10 μM recombinant α-syn monomer with phenolic compounds utilizing a paradigm of constant shaking at physiologic temperatures for 24 h which, in prior experiments, resulted in robust fibril formation (Ono et al., 2012). We then applied varying concentrations of aggregate mixture with α-syn concentration ranging from 2 to 666 nM to α-syn -A53T-CFP/YFP cells and incubated for a 24 and 72 h period. Phenolic acid inhibition of the α-syn aggregation was then quantified with flow cytometry via measurement of FRET signal (% positive cells, which is indicative of the formation and accumulation of intracellular α-syn aggregates).

**FIGURE 1:**
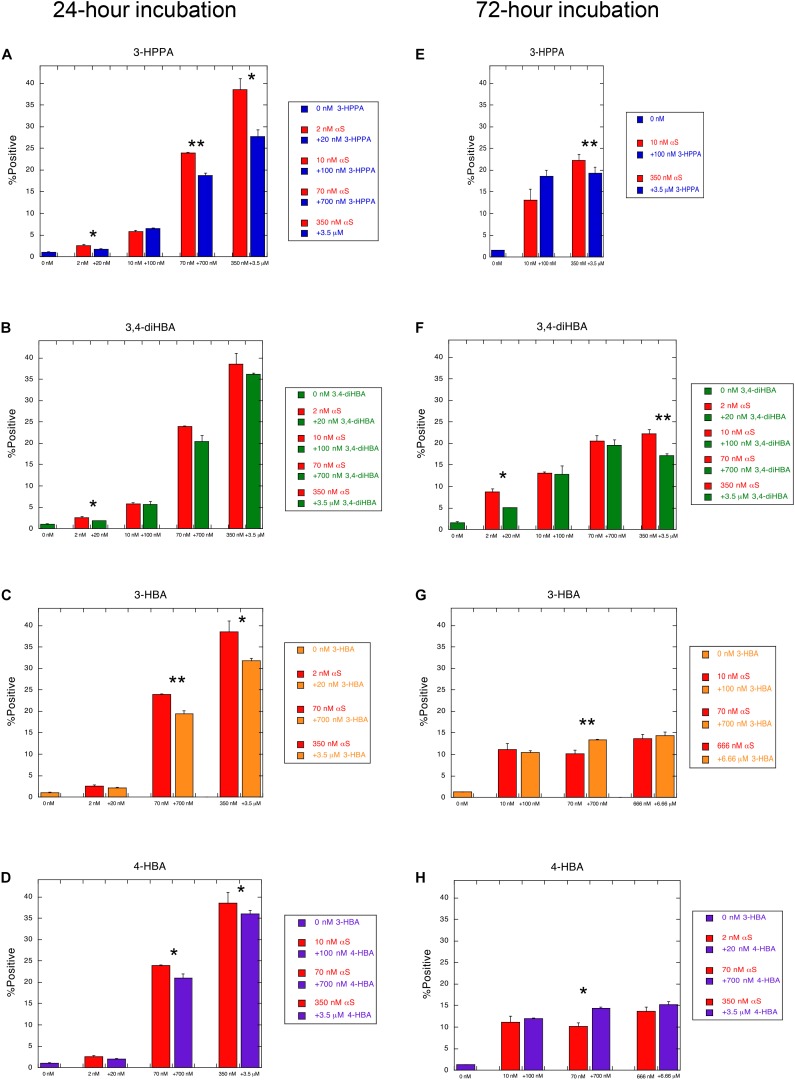
Phenolic acids inhibit formation of aggregation-prone forms of α-syn. α-syn-A53T-CFP/YFP cells were assessed for% positive FRET signal after a 24 h incubation with varying concentrations of α-syn aggregated in the presence of phenolic acid compounds **(A)** 3-HPPA **(B)** 3,4-diHBA **(C)** 3-HBA and **(D)** 4-HBA. FRET signal was quantified in a second set of α-syn-A53T-CFP/YFP cells after a 72 h incubation in the presence of varying concentrations of α-syn exposed to **(E)** 3-HPPA **(F)** 3,4 diHBA **(G)** 3-HBA and **(H)** 4-HBA during the aggregation phase. Data from each subject is presented as a Mean ± SEM from four replicate assays. Experimental replicates were 1–2 (*p < 0.05, **p < 0.01, Unpaired *t-*test compared to α-syn aggregated without exposure to phenolic acid).

We observed that phenolic compounds 3-HPPA, 3,4-diHBA 3-HBA, and 4-HBA inhibited the formation of α-syn species capable of seeding aggregation of α-syn within biosensor cells at 1:10 ratio. This was most apparent at an early (24 h) timepoint of incubation in the cell-based assay (Figures 1A–D). Compound 3-HPPA inhibited generation of α-syn intracellular aggregate formation over a range of seeded concentrations (2, 70, and 350 nM) (Figure 1A). 3-HBA and 4-HBA similarly showed inhibitory activity against α-syn aggregation, especially at higher concentrations of α-syn (70 and 350 nM) at the 24 h timepoint (Figures 1C,D). 3,4-diHBA was less effective at suppressing the generation of α-syn seed competent forms (Figure 1B).

The generation of intracellular aggregates was also quantified by FRET at the 72 h timepoint to determine if increased incubation time resulted in change in aggregate formation. Compound 3-HPPA had persistent reduction of aggregation at this timepoint at a high (350 nM) α-syn concentration (Figure 1E). Compound 3,4-di-HBA did demonstrate significant inhibition of aggregation-prone α-syn forms at the 2 and 350 nM concentrations at the 72 h timepoint only (Figure 1F), Other samples tested, 3-HBA and 4-HBA, did not have significant impact on FRET seeding activity, although a slight elevation in aggregation was seen at the 70 nM seeded concentration only (Figures 1G,H).

### α-Syn Aggregation Induced by Brain Samples Is Inhibited by Specific Gut-Modified Phenolic Compounds

Recombinant α-syn fibrils generated from monomer by a high-speed shaking paradigms may not necessarily have physiologic relevance to forms generated in the brain during conditions of slow progressive neurodegeneration seen in PD and the related synucleinopathy, MSA. However, new cyclic amplification assays, such as real-time quaking-induced conversion and protein misfolding cyclic amplification have successfully utilized this method in combination with thioflavin T (ThT) for detection of α-syn prone aggregating forms in CSF and brain tissue of patients with synucleinopathies (Herva et al., 2014; Fairfoul et al., 2016; Shahnawaz et al., 2017).

To determine whether polyphenolic compounds would have the ability to inhibit α-syn seeding activity as generated by forms found in PD and MSA brain, we incubated polyphenolic compounds with insoluble brain fractions (generated by serial buffer and detergent extraction) in a similar monomeric shaking paradigm. We utilized brain samples with neuropathologically-confirmed diagnoses of PD or MSA. Insoluble fractions of these brains could be classified as either “low seeders” or “high seeders” given prior assessment via FRET (Yamasaki et al., 2019). Reaction mixture was exposed to biosensor cells in the presence of lipofectamine and aggregate load was quantified at 24 h.

There was no significant inhibition of formation of aggregate generating forms from “high seeder” type brain insoluble extracts from PD and MSA (Table 2). However, it is possible that “high seeder” brain fractions may contain a more aggregate-prone form of α-syn than “low seeder” counterparts, and thus outstrip the ability of phenolic compounds to inhibit aggregate formation. We therefore evaluated “low seeder” forms to see if phenolic compounds could have inhibitory activity on aggregate formation generated by these neuropathologically-characterized brain fractions. Strikingly, both 3,4-diHBA and 3-HPPA significantly inhibited generation of aggregate-prone forms for both MSA and PD “low seeder” samples (Table 2). A subsequent expansion of this testing to a limited number of “low seeder” brains also showed the ability to inhibit aggregate formation in both PD and MSA although the sample of PD2 (AC area) and MSA1 (CB area) showed inconsistency (Table 2).

**TABLE 2:** Phenolic acids inhibit formation of α-syn aggregates induced by exposure to PD and MSA brain extracts with low potency for seed formation.

**Phenotype**	**Brain region**	**Control 300 nM**	**300 nM α-syn**	**3-HPPA 3 μM**	**3,4-diHBA 3 μM**
		**High seeders (% FRET positive cells)**			
MSA 4	BG	2*36.6 ± 1.6	36.1 ± 0.7	37.8 ± 1.4	35.5 ± 1.1
PD 5	AC		16.0 ± 0.8	37.3 ± 1.6	36.6 ± 1.7
		**Low seeders (% FRET positive cells)**			
PD 1	AC	4*27.0 ± 1.0	21.8 ± 0.5	14.2 ± 0.5**	11.4 ± 0.5**
PD 2	AC		32.6 ± 0.9	31.1 ± 1.7	32.9 ± 2.1
MSA 1	BG		24.4 ± 1.0	16.3 ± 0.7**	16.1 ± 0.4**
MSA 1	CB		22.7 ± 0.8	32.1 ± 1.6	34.6 ± 1.3

PD, Parkinson’s disease; MSA, multiple system atrophy; AC, anterior cingulate; BG, basal ganglia; CB, cerebellum. Each sample contains one individual with four technical replicates ± SEM. ** p < 0.01, by one-way ANOVA with post-hoc Dunnett test compared to the brain extracts without exposure to phenolic acid.

## Discussion

Recent studies support the idea that abnormal proteins are amplified by the same mechanism as prions and are transmitted between cells in major neurodegenerative diseases, including PD and AD (Frost and Diamond, 2010; Guo and Lee, 2014). Experimental evidence for this cell-to-cell transmission of pathological α-syn also includes the results of transplantation of embryonic dopaminergic neurons into PD patients (Li et al., 2008; Li et al., 2016). These reports highlighted the possibility that misfolded α-syn accumulated in PD patients was transmitted from the host brain cells to the grafts, resulting in the appearance of Lewy pathologies in the transplanted cells. Seed-dependent aggregation of α-syn is also observed in various kinds of cultured cells and primary-cultured neurons (Desplats et al., 2009; Luk et al., 2009). Introduction of synthetic α-syn fibrils into human neuroblastoma SH-SY5Y cells expressing α-syn induced intracellular accumulation of phosphorylated α-syn, which is similar to abnormal α-syn deposits in the brains of patients with LB diseases (Nonaka et al., 2010). Furthermore, these α-syn aggregates bound thioflavin dye, which means β-sheet structure. Immuno-electron microscopy revealed that Sarkosyl-insoluble fractions extracted from SH-SY5Y cells into which fibrils had been introduced contained abundant fibrillar structures labeled with an antibody specific for α-syn phosphorylated at Ser129 (Nonaka et al., 2010). Thus, nucleation and prion-like seed-templated amplification of abnormal α-syn aggregates are reproduced *in vitro*.

**FIGURE 2:**
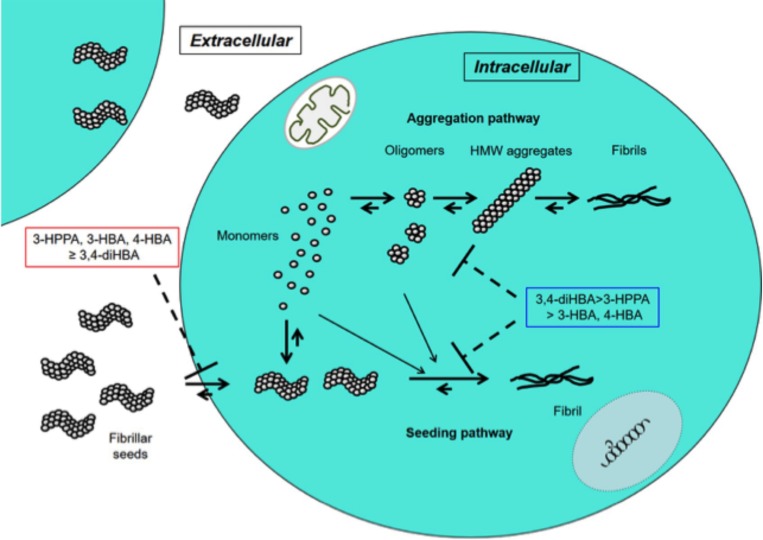
Inhibitory effects of 3-HPPA, 3,4-diHBA, 3-HBA, and 4-HBA on intracellular α-syn fibril formation. In the cell, monomer of α-syn may aggregate to form intermediate aggregates such as oligomers, HMW aggregates and finally fibrils as Lewy body. On the other hand, fibrillar seeds of α-syn entering from outside the cell exert as seeds to form α-syn fibrils within the cell. 3-HPPA may prevent not only penetration of fibrillar seeds of α-syn into cells but also aggregation and seeding pathways of α-syn. 3,4-di-HBA may mainly prevent aggregation and seeding pathways of α-syn.

In this study 3-HPPA and 3,4-diHBA, and to a lesser extent 3-HBA and 4-HBA demonstrated the ability to partially inhibit generation of aggregate prone forms from α-syn monomer. Compounds 3-HPPA, 3-HBA and 4-HBA all showed a robust level of inhibition of α-syn seeding activity across multiple seeded concentrations of the reaction mixture. This was detected at an early incubation timepoint. Only 3-HPPA showed consistency across multiple timepoints. The loss of effectiveness of 3-HBA and 4-HBA, and even slight loss of signal in 3-HPPA at the 72 h timepoint could be due to several factors. There is an overall reduction in % positive cells detected by FRET at most of the different α-syn concentrations at 72 h, implying that biosensor cells without aggregates continued to propagate, while cells with aggregates did not efficiently pass the aggregates on to their daughter cells. Another explanation is that there was decreased viability of cells with inclusions by the 72 h timepoint. A final possible explanation is the difference of the inhibitory effect of these compounds on penetration of fibrillar seeds into cells. Considering aggregate data for the 24 and 72 h, 3-HPPA may prevent not only penetration of fibrillar seeds of α-syn into cells but also interfere with aggregation and seeding pathways of α-syn, whereas 3,4-di-HBA may mainly prevent aggregation and seeding pathways of α-syn (Figure 2).

In previous work, we demonstrated that 3-HPPA, 3,4-diHBA, and 3-HBA are capable of attenuating the assembly of monomeric synthetic α-syn into fibrils as detected by ThT signal (Ho et al., 2019). Interestingly, with the cell-based method of α-syn aggregation used in these experiments, we observed that only 3-HPPA, 3-HBA, and 4-HBA were able to provide some level of attenuation of the assembly the synthetic α-syn into forms capable of supporting the induction of intracellular α-syn aggregation. Phenolic acid 3,4-diHBA was less consistent in this respect. These contrasting results could be due to differences in assay detection substrate. ThT will detect a fibrillar β-pleated sheet formation, which is thought to be a late-stage form in the process of aggregation of amyloid proteins (Ono et al., 2008; Soto and Pritzkow, 2018). Small oligomeric (non-fibrillar) forms are also efficient in inducing aggregation of monomeric α-syn (Ono et al., 2011; Chen et al., 2015). While the ThT assay therefore, is useful for detecting the end-product of aggregation, smaller forms with potency for aggregation may be more sensitively detected by cell-based assays or other methods that specifically target oligomers.

One explanation for some inconsistency with seeding response may be due to the potency of fibril formation as initiated by a high-speed shaking paradigm. Although this is a popular paradigm for creation of pre-formed fibrils, formation of these aggregated forms does not mimic physiologic conditions. The species formed may have different conformations or aggregation potential than physiologically-derived α-syn aggregation-prone types and thus may be less inhibited by phenolic compounds.

**FIGURE 3:**
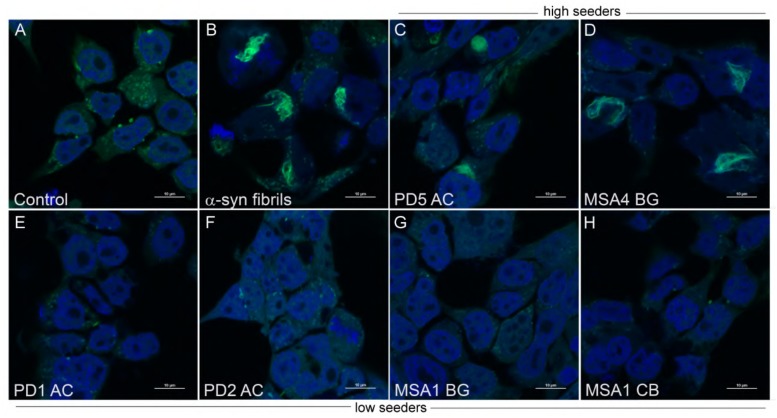
Aggregate morphology within α-syn CFP/YFP cells exposed to brain insoluble fractions. Cells exposed to α-syn fibrils develop intracellular aggregates which differ in morphology from aggregates formed by exposure to insoluble brain extract from PD and MSA brain. **(A)** Control CFP/YFP cells only (no exogenous α-syn) **(B)** Cells exposed to 10 nM preformed α-syn fibrils **(C,D)** PD and MSA insoluble brain fractions utilized in “high seeder” experiments form aggregates with diverse morphology when exposed to α-syn CFP/YFP cells **(E–H)** PD and MSA insoluble brain fractions utilized in “low seeder” experiments did not demonstrate robust aggregate formation when introduced into α-syn CFP/YFP cells. These images were adapted from our previous paper (Yamasaki et al., 2019). Scale bars indicate 10 μm.

To enhance physiologic relevance, we tested the ability of phenolic compounds to inhibit the generation of aggregate-prone forms of α-syn from a limited number of PD and MSA brain extracts. Both 3,4-di-HBA and 3-HPPA demonstrated the ability to inhibit seeding activity from PD and MSA brain. The inconsistency of seeding inhibition by phenolic acids for “high seeder” and “low seeder” MSA and PD brain could be related to the affinity of the proteopathic form of α-syn to form aggregates. There is some indication that different synucleinopathies may harbor different forms or “strains” of α-syn (Bousset et al., 2013; Guo et al., 2013; Watts et al., 2014; Peelaerts et al., 2015; Peng et al., 2018; Candelise et al., 2019; Yamasaki et al., 2019). It is possible that “low seeder” vs. “high seeder” PD and MSA brains contain diverse strains with varied propensity for aggregate formation and that phenolic acid inhibition of aggregation relies on the underlying conformation of the proteopathic α-syn form (Figure 3). Ongoing studies are investigating specific forms of aggregated α-syn that are generated by incubation of α-syn alone, or by incubation in the presence of MSA and PD brain extracts. Nonetheless, our observation suggests that select brain-accumulating phenolic acids derived from gut microbiota metabolism of dietary polyphenols may interfere with α-syn spreading by attenuating the generation of oligomeric α-syn forms capable of inducing further misfolded α-syn. Recent Cryo-electron microscopy analysis has shown that the fibril structure of α-syn differs *in vitro* and *in vivo* (Meade et al., 2019). Furthermore, it has been pointed out that the fibril structure may be different depending on the post-translational modification (Meade et al., 2019). Further structural studies are essential not only to determine whether α-syn expressing in monoclonal biosensor cell has a sensory modification, but also to reveal the structural destabilization of intracellular α-syn aggregates by phenoloic compounds. Outcomes from our studies suggest interactions between gut microbiome and certain dietary factors may form the basis for effective therapies that modulate pathologic α-syn propagation. Collectively, our findings provide the basis for future development of probiotic, prebiotic, or symbiotic approaches for modulating the onset and/or progression of PD, MSA and other synucleinopathies.

## Data Availability statement

The datasets generated for this study are available on request to the corresponding author.

## Ethics statement

Ethical review and approval was not required for the study on human participants in accordance with the local legislation and institutional requirements. Written informed consent from the participants was not required to participate in this study in accordance with the national legislation and the institutional requirements.

## Author Contributions

GP, LH, KO, and TY conceived of the experiments and analyzed the data. TY performed the experiments. GP, KO, and TY wrote the manuscript.

## Conflict of Interest

The authors declare that the research was conducted in the absence of any commercial or financial relationships that could be construed as a potential conflict of interest.
