# Supplementary figures and images for: Gut Microbiome-Modified Polyphenolic Compounds Inhibit α-Synuclein Seeding and Spreading in α-Synucleinopathies
Source: Front Neurosci. 2020 May 4;14:398. doi: 10.3389/fnins.2020.00398 (PMC7212829; doi:10.3389/fnins.2020.00398)

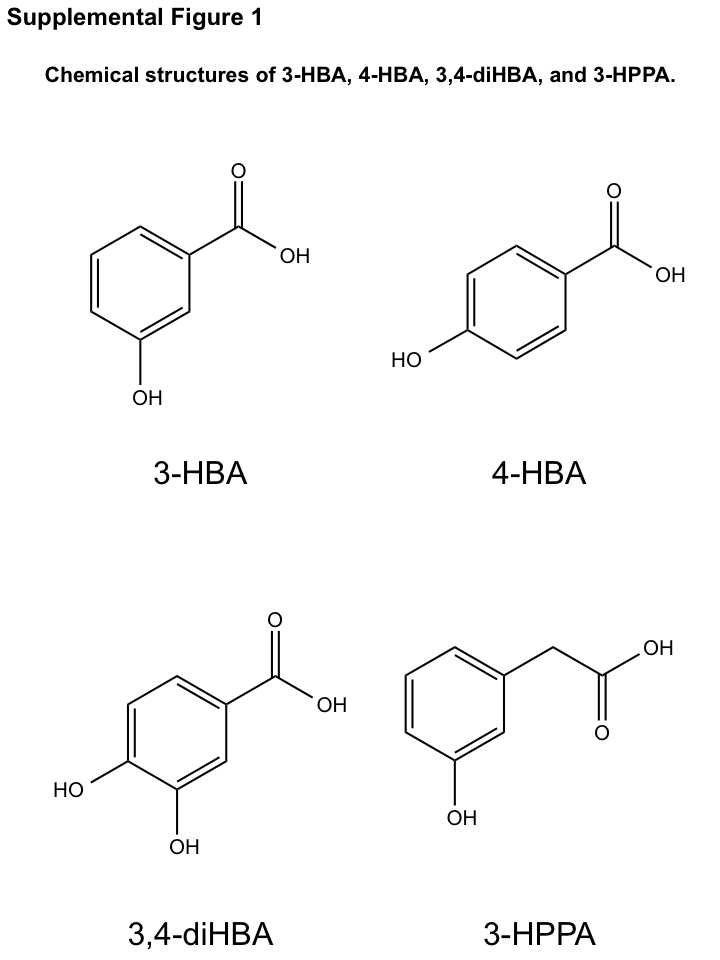

Supplement: Supplementary file 1 [file Image_1.TIFF]
